# Supplementary material for: Pharmacological reversal of synaptic and network pathology in human MECP2‐KO neurons and cortical organoids
Source: EMBO Mol Med. 2020 Dec 8;13(1):e12523. doi: 10.15252/emmm.202012523 (PMC7799367; doi:10.15252/emmm.202012523)
Supplement: Supplementary file 1 — Expanded View Figures PDF [file EMMM-13-e12523-s001.pdf]

Expanded View Figures

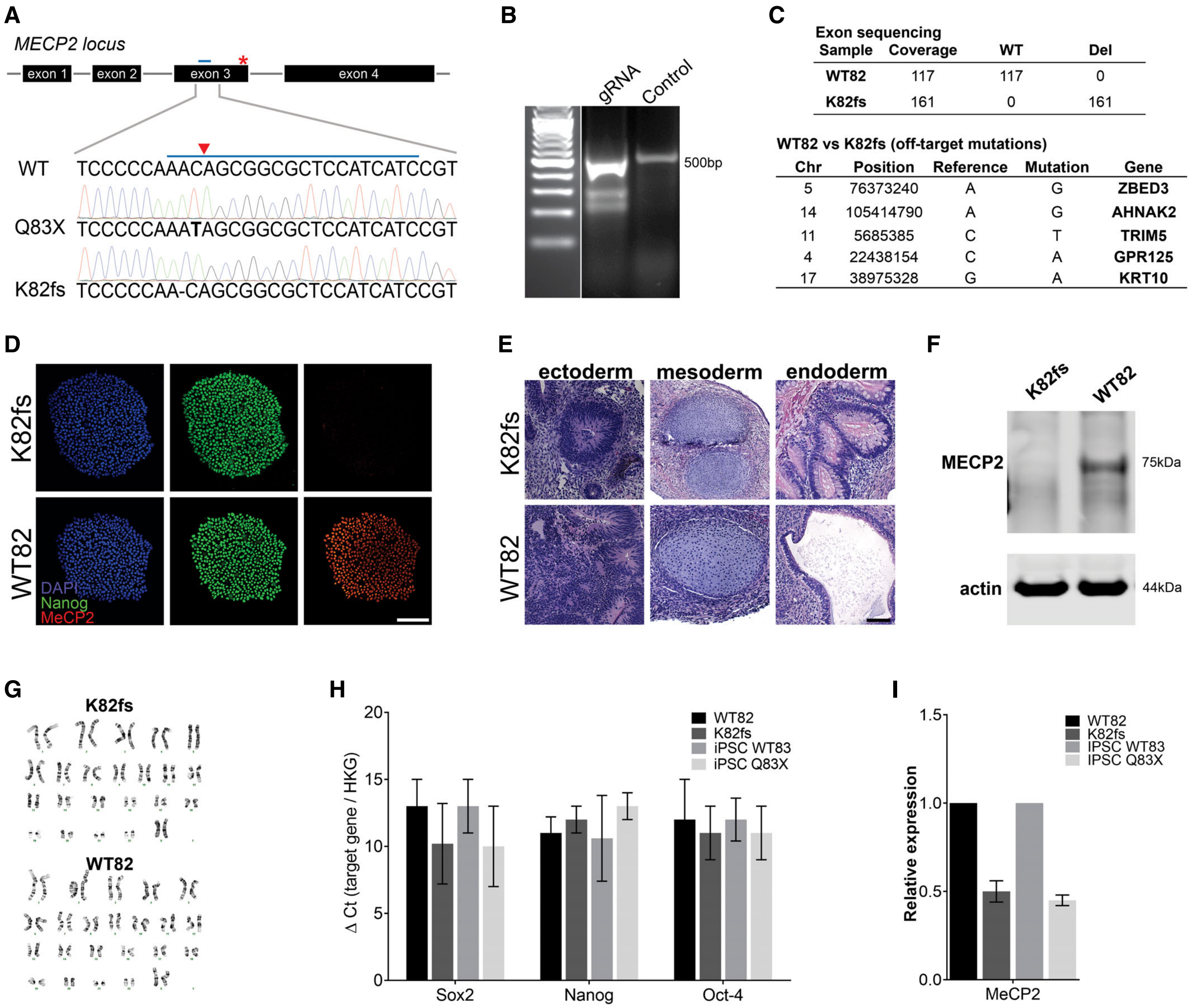

Figure EV1. Generation of *MECP2*-KO pluripotent stem cells.

A Mutations resulting in loss of functional MeCP2 protein shown by Sanger sequencing. CRISPR guide RNA is depicted as a blue bar over WT sequence and the cut site as a red triangle.

B CRISPR guide RNA to generate K82 frameshift mutation.

C Identification of off-target mutations by exome sequencing.

D–I *MECP2*-KO was successfully generated and did not affect pluripotency, as confirmed by immunochemistry (D), teratoma assay (E), Western blot (F), karyotype (G), and RT–qPCR (H, I). Experiments were performed in triplicate; data are presented as mean ± s.e.m. Scale bar = 200 μm.

© 2020 The Authors

Figure EV2. *MECP2*-KO neurons exhibit defects in spine-like morphology.

A Schematic showing neuronal differentiation from human PSCs. Scale bar = 200 μm.
B Neuronal markers immunostaining of 28-days *MECP2*-KO and control neurons. Scale bar = 50 μm.
C No difference in the resultant proportions of neurons or glia was observed between 28-days *MECP2*-KO and control neural differentiations. Similar results were observed for the proportions of glutamatergic and GABAergic neurons. Experiments were performed in triplicates.
D 28-days *MECP2*-KO neurons had decreased soma area and spine number (WT83/Q83X cell lines were used; $N = 13$ neurons per genotype, Student's t-test, $t_{24} = 5.03$, ***$P < 0.0001$). Left: representative images of Syn::GFP + neurons; Right: neuronal feature quantification. Scale bar = 20 μm.
E Observation over time showed decreased spine stability (*$P < 0.01$), but spine motility ($P = 0.17$), spine formation ($P = 0.26$), and spine length ($P = 0.28$) were unchanged in *MECP2*-KO neurons. Scale bar = 5 μm. WT83/Q83X cell lines were used, N = 10-15 neurons/condition. Analyses were two-sided Student's t-tests. Top: representative images of neuronal spine dynamics at 5-min intervals; arrows denote representative spines. Bottom: quantification of features of neuronal spine dynamics.

Data information: Data are presented as mean ± s.e.m.

 © 2020 The Authors

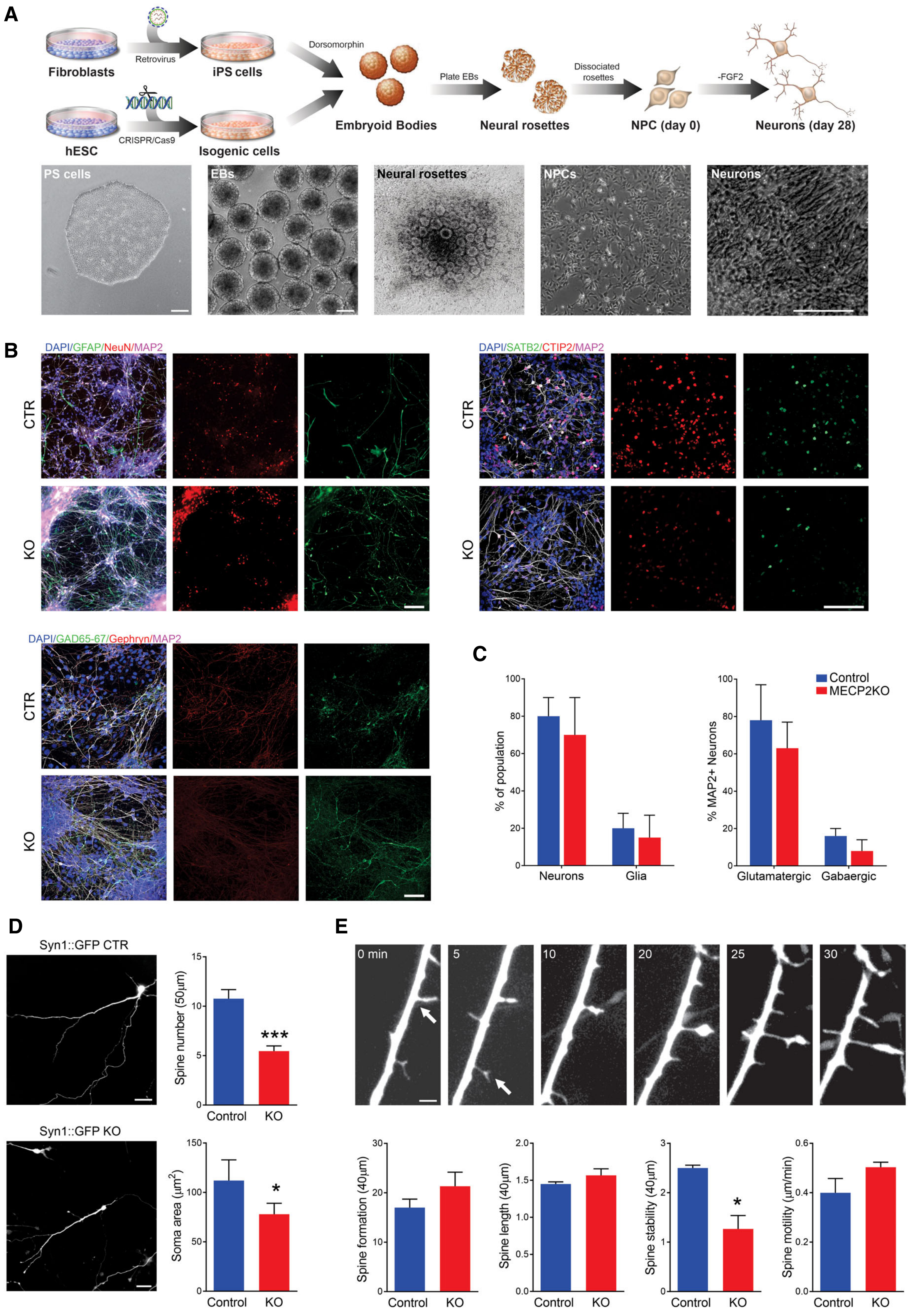
A
Fibroblasts
Retrovirus
iPS cells
hESC
CRISPR/Cas9
Isogenic cells
Dorsomorphin
Embryoid Bodies
Plate EBs
Neural rosettes
Dissociated rosettes
NPC (day 0)
-FGF2
Neurons (day 28)
PS cells
EBs
Neural rosettes
NPCs
Neurons
B
DAPI/GFAP/NeuN/MAP2
DAPI/SATB2/CTIP2/MAP2
CTR
KO
DAPI/GAD65-67/Gephryn/MAP2
C
% of population
Neurons
Glia
% MAP2+ Neurons
Glutamatergic
Gabaergic
Control
MECP2KO
D
Syn1::GFP CTR
Syn1::GFP KO
Spine number (50µm)
Soma area (µm2)
Control
KO
E
0 min
5
10
20
25
30
Spine formation (40µm)
Spine length (40µm)
Spine stability (40µm)
Spine motility (µm/min)

Figure EV2.

Figure EV3. RT–PCR array and single-cell RT–qPCR analyses.

A, B RT–PCR array revealed that differences in genetic expression showed variation between control and *MECP2*-KO NPCs and neurons, as depicted on volcano plots (A) and heatmap (B). Each heatmap column is one gene.

C–K Single-cell RT–qPCR analysis likewise showed differences in gene expression throughout neuronal differentiation due to *MECP2*-KO that appear to involve synaptic function. 28-days WT83/Q83X and WT82/K82fs neurons were used. Principal component analysis (C) of control and *MECP2*-KO NPCs (day 0 of same cell lines) and comparative expression of select genes between genotypes (D). Principal component analysis shows differences in expression between NPCs and neurons within each genotype (E) and differences in neuronal gene expression between genotypes (F). Heatmap shows differences in expression of particular genes (columns; G), and schematic denotes single-cell qPCR of individual markers (H). Number (H) and graphical depiction (J) of differentially expressed genes between genotypes. Table presents differentially expressed genes (K).

 © 2020 The Authors

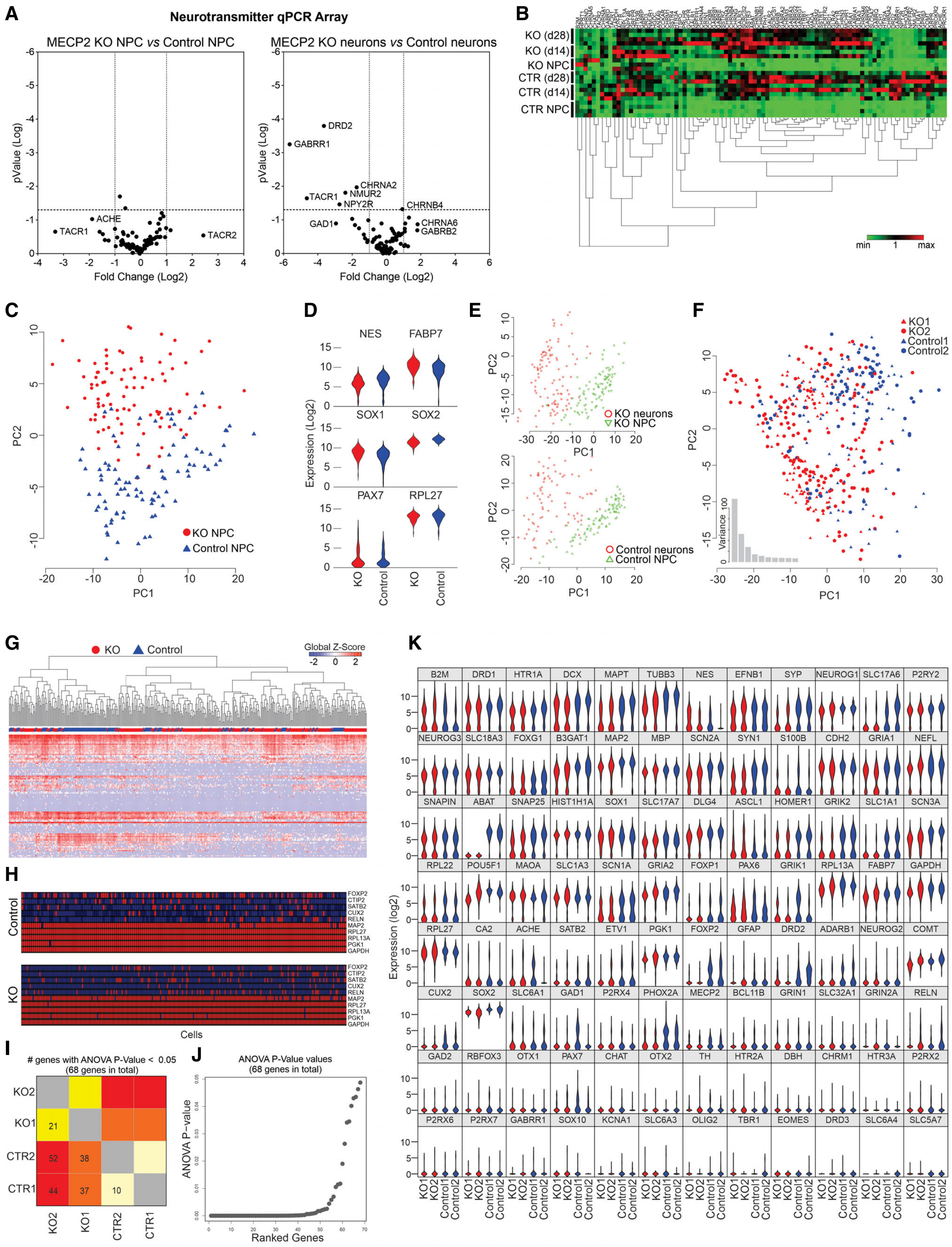
A
Neurotransmitter qPCR Array
MECP2 KO NPC vs Control NPC
MECP2 KO neurons vs Control neurons
pValue (Log)
Fold Change (Log2)
B
KO (d28)
KO (d14)
KO NPC
CTR (d28)
CTR (d14)
CTR NPC
min
1
max
C
KO NPC
Control NPC
PC1
PC2
D
Expression (Log2)
E
KO neurons
KO NPC
Control neurons
Control NPC
F
KO1
KO2
Control1
Control2
Variance
G
KO
Control
Global Z-Score
H
Control
KO
Cells
I
genes with ANOVA P-Value < 0.05
(68 genes in total)
J
ANOVA P-Value values
(68 genes in total)
ANOVA P−value
Ranked Genes
K
Expression (log2)

Figure EV3.

Figure EV4. Drug treatment of *MECP2*-KO and control neurons.

A Representative Western blot images after *MECP2*-KO drug treatment. Samples run at the same time in different gels.
B Analysis of treatment effect on quantities of the astrocytic marker GFAP and neuronal marker MAP2. Experiments were performed in triplicate.
C Full depiction of synaptic puncta following drug treatment. Scale bar = 5 μm.
D Synaptic puncta co-localization following drug treatment of 6-week control neurons (WT83/Q83X cell lines were used; $N = 8$ neurons per condition; one-way ANOVA, $F_{6,49} = 10.94$, *$P < 0.05$). ** $P < 0.01$, *** $P < 0.001$. *Z* scores of control untreated vs. compound: Nefiracetam = 1.010; PHA 543613 = 0.282; Acamprosate = 0.175; GR73632 = 1.010; IGF-1 = 3.231; Hyperforin = 2.639).
E, F Calcium imaging showed increasing spike frequency with time (E) that was tractable to manipulators of synaptic function (F). Experiments were performed in triplicate.
G Representative image of MEA activity of control and *MECP2*-KO neurons.

Data information: Data are presented as mean ± s.e.m.

 © 2020 The Authors

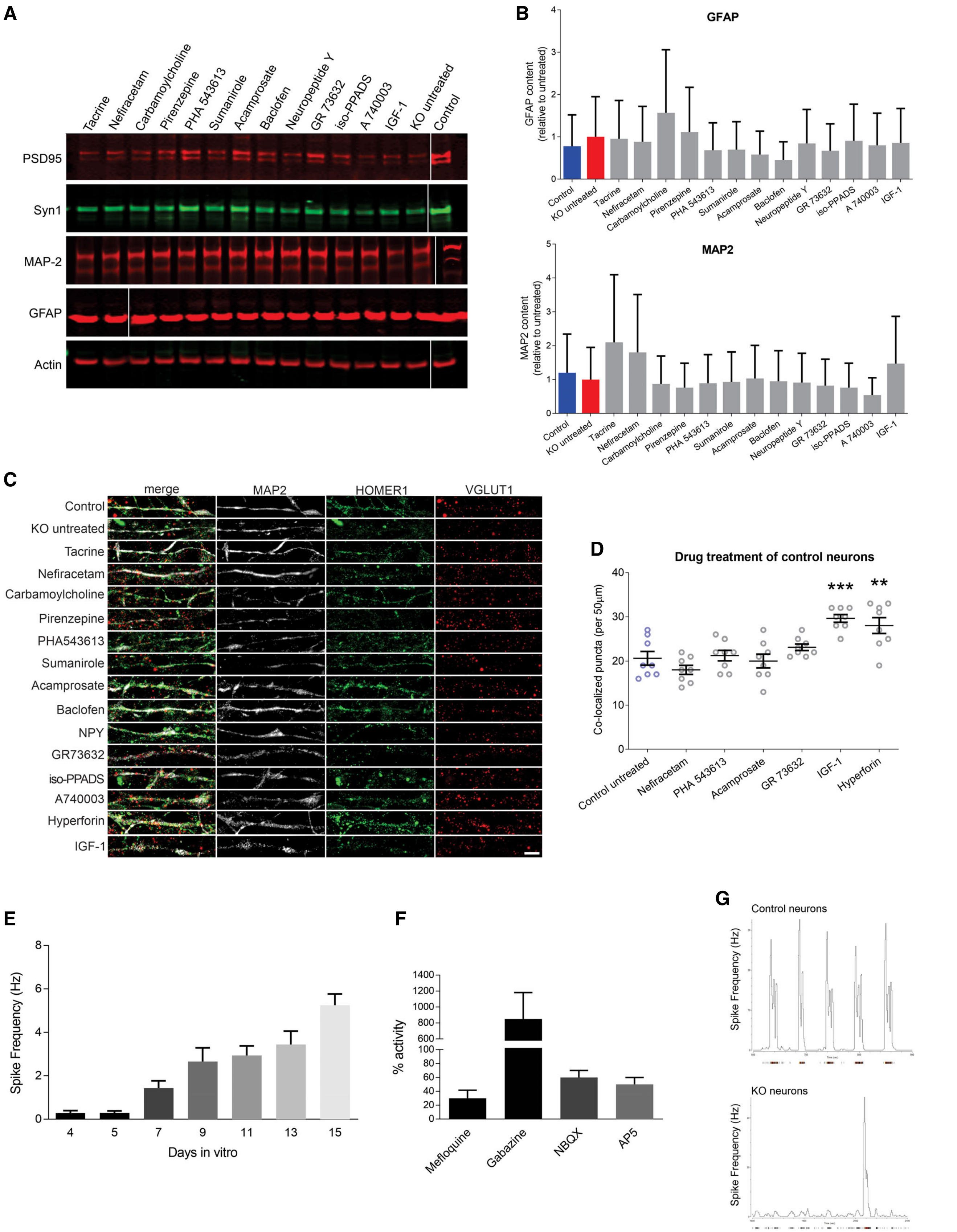
A
Tacrine
Nefiracetam
Carbamoylcholine
Pirenzepine
PHA 543613
Sumanirole
Acamprosate
Baclofen
Neuropeptide Y
GR 73632
iso-PPADS
A 740003
IGF-1
KO untreated
Control
PSD95
Syn1
MAP-2
GFAP
Actin
B
GFAP
GFAP content (relative to untreated)
MAP2
MAP2 content (relative to untreated)
C
merge
MAP2
HOMER1
VGLUT1
KO untreated
NPY
D
Drug treatment of control neurons
Co-localized puncta (per 50μm)
Control untreated
Hyperforin
E
Spike Frequency (Hz)
Days in vitro
F
% activity
Mefloquine
Gabazine
NBQX
AP5
G
Control neurons
KO neurons

Figure EV4.

Figure EV5. Cellular and molecular characterization of human cortical organoids.

A Schematic of the protocol used to generate cortical organoids. Scale bar = 200 μm.

B Reproducibility of organoid size at 3 weeks of maturation (*N* = *20* independent experiment, 7 different cell lines).

C, D Gene expression of specific neurotransmission markers of treated vs. untreated cortical organoids (*N* = 8 samples of treated organoids and 8 samples of control organoids, ~10–15 organoids pooled per sample). Boxplot: center band of the box is median $\log_2$ normalized expression and edges are 25th and 75th percentiles; whiskers are minimum and maximum $\log_2$ normalized expression values.

E, F Representative images of quantification of Synapsin1 puncta. Scale bar = 50 μm. SYN1 puncta mask was made using ImageJ to access the number and area of the puncta.

G Nefiracetam and PHA 543613 treatment did not rescue *MECP2*-KO neuronal morphology, for either the number of neurites (Kruskal–Wallis test, $P = 0.011$; Dunn's multiple comparisons test vs. KO untreated: control, $^{**}P = 0.01$ and $Z = 3.13$; Nefiracetam, $P = 0.49$ and $Z = 1.74$; PHA 543613, $P > 0.99$ and $Z = 0.87$; WT83/Q83X and WT82/K82fs cell lines; N = 5–10 neurons per condition) or the total neurite length (one-way ANOVA, $F_{3,21} = 9.62$, $P = 0.0003$; Dunnett's multiple comparisons test vs. KO untreated: control, $^{**}P = 0.002$ and $Z = 3.08$; Nefiracetam, $P = 0.89$ and $Z = 0.64$; PHA 543613, $P = 0.99$ and $Z = 0.17$; WT83/Q83X and WT82/K82fs cell lines; N = 5–10 neurons per condition) and, nearly uniformly, did not rescue nuclei size in neurons (one-way ANOVA, $F_{3,221} = 26.04$, $P < 0.0001$; Dunnett's multiple comparisons test vs. KO untreated: control, $^{***}P < 0.0001$ and $Z = 6.37$; Nefiracetam, $P = 0.91$ and $Z = 0.39$; PHA 543613, $P = 0.19$ and $Z = 1.74$; WT83/Q83X and WT82/K82fs cell lines; N = 45 nuclei per condition), neurospheres (one-way ANOVA, $F_{5,264} = 5.579$, $P < 0.0001$; Dunnett's multiple comparisons test vs. CTR/KO untreated: control, $^{***}P < 0.0001$ and $Z = 3.782$; KO untreated, $P = 0.51$ and $Z = 1.066$; Nefiracetam, $P = 0.23$ and $Z = 1.35$; PHA 543613, $P = 0.99$ and $Z = 0.05$; Nefi + PHA, $P = 0.24$ and $Z = 1.012$; WT83/Q83X cell lines; N = 45 nuclei per condition), or cortical organoids (one-way ANOVA, $F_{3,281} = 6.594$, $P = 0.0003$; Dunnett's multiple comparisons test vs. KO untreated: control, $^{**}P = 0.009$ and $Z = 2.38$; Nefiracetam, $^{**}P = 0.01$ and $Z = 3.05$; PHA 543613, $P = 0.98$ and $Z = 0.30$; WT83/Q83X and WT82/K82fs cell lines; N = 45-90 nuclei per condition). Spine density was rescued or partially rescued by the compounds (one-way ANOVA, $F_{3,63} = 8.449$, $P < 0.0001$; Dunnett's multiple comparisons test vs. KO untreated: control, $^{***}P < 0.0001$ and $Z = 4.35$; Nefiracetam, $P = 0.16$ and $Z = 2.01$; PHA 543613, $^{***}P = 0.001$ and $Z = 3.24$; WT83/Q83X and WT82/K82fs cell lines; N = 11–28 neurons per condition). Note that statistical comparisons are presented relative to KO untreated or, for mosaic neurospheres, CTR/KO untreated.

Data information: Data are presented as mean ± s.e.m.

 © 2020 The Authors

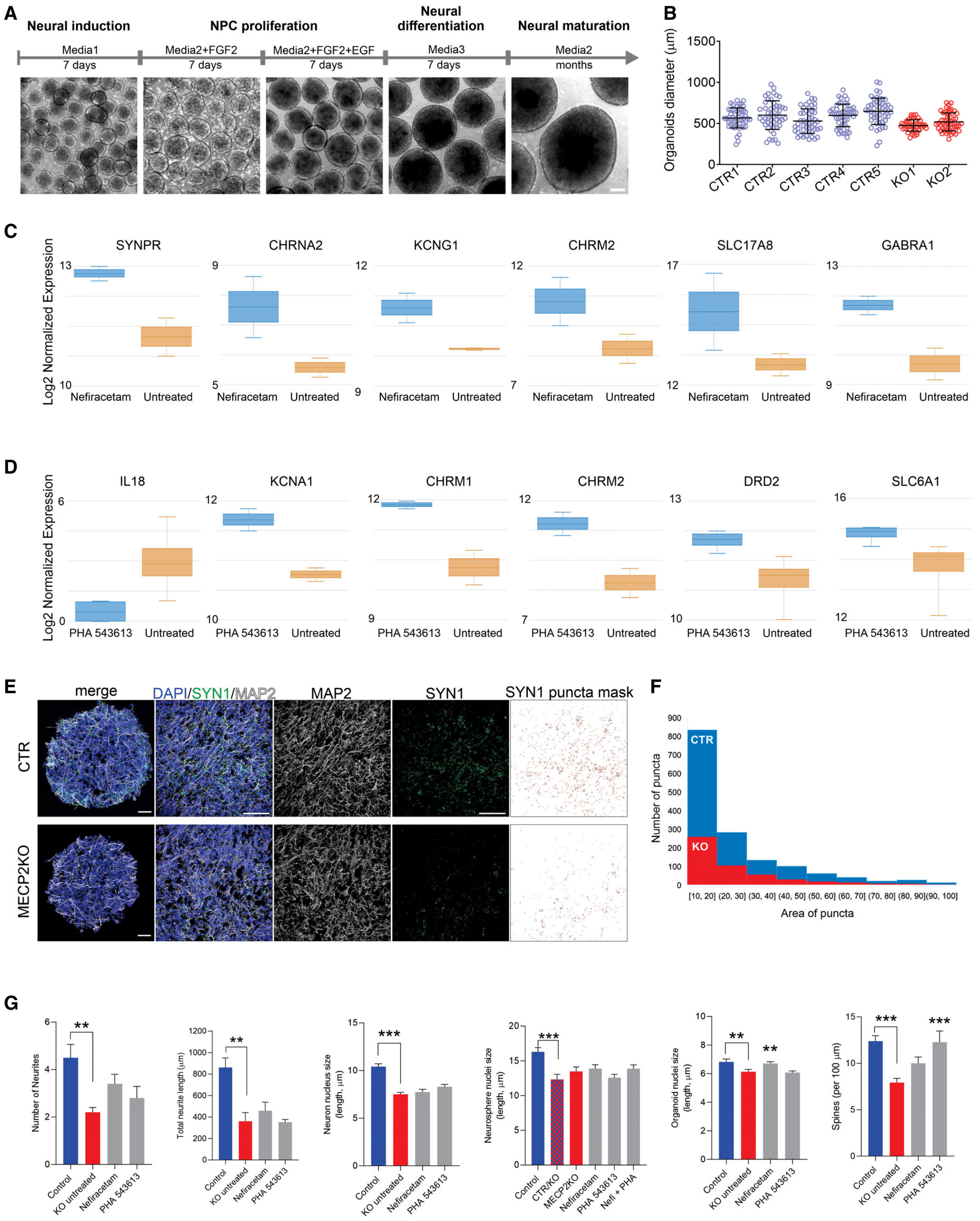
A
Neural induction
NPC proliferation
Neural differentiation
Neural maturation
Media1 7 days
Media2+FGF2 7 days
Media2+FGF2+EGF 7 days
Media3 7 days
Media2 months
B
Organoids diameter (μm)
CTR1 CTR2 CTR3 CTR4 CTR5 KO1 KO2
C
Log2 Normalized Expression
SYNPR CHRNA2 KCNG1 CHRM2 SLC17A8 GABRA1
Nefiracetam Untreated
D
Log2 Normalized Expression
IL18 KCNA1 CHRM1 CHRM2 DRD2 SLC6A1
PHA 543613 Untreated
E
merge
DAPI/SYN1/MAP2
MAP2
SYN1
SYN1 puncta mask
CTR
MECP2KO
F
Number of puncta
CTR
KO
Area of puncta
G
Number of Neurites
Total neurite length (μm)
Neuron nucleus size (length, μm)
Neurosphere nuclei size (length, μm)
Organoid nuclei size (length, μm)
Spines (per 100 μm)
Control
KO untreated
Nefiracetam
PHA 543613
CTR/KO
MECP2KO
Nefi + PHA

Figure EV5.
